# Matrix Metalloproteinases as a Pleiotropic Biomarker in Medicine and Biology

**DOI:** 10.1155/2016/9275204

**Published:** 2016-11-09

**Authors:** Jacek Kurzepa, Fatma M. El-Demerdash, Massimiliano Castellazzi

**Affiliations:** ^1^Department of Medical Chemistry, Medical University of Lublin, Chodzki 4a, 20-093 Lublin, Poland; ^2^Environmental Studies Department, Institute of Graduate Studies and Research, University of Alexandria, 163 Horrya Av., P.O. Box 832, El Shatby, Alexandria, Egypt; ^3^Department of Biomedical and Specialist Surgical Sciences, Section of Neurological, Psychiatric and Psychological Sciences, University of Ferrara, 44124 Ferrara, Italy

The group of matrix metalloproteinases (MMPs), calcium- and zinc-dependent proteolytic enzymes, is responsible for extracellular protein degradation. Acting together, supported by intracellular processes they are able to digest any physiological extracellular protein. However, the biochemistry of extracellular matrix (ECM) is very complex, and proteolytic enzymes located in this compartment exert numerous pleiotropic effects beyond the characteristic for the degradation of structural elements. Therefore, MMPs are involved into several physiological and pathological processes [1].

Because of the ECM components' ability to model, as well as the influence on the activity of some biologically active compounds such as tumor necrosis factor *α*, chemokine CXCL-8, and transforming growth factor *β*, MMPs affect the pathogenesis of numerous diseases, mostly primarily associated with inflammation [2]. Therefore, the elevated level of particular MMP cannot be associated with failure of specific organ or tissue. In that case the MMPs can be biomarkers of disease? MMPs are sensitive and easily measurable, but due to their prevalence they are not specific for any tissue. For example, MMP-9 serum level is elevated in patients with relapsing remitting and secondary progressive multiple sclerosis (MS) compared to controls [3] and the MMP-9/TIMP-1 ratio may predict magnetic resonance image (MRI) activity during interferon-beta therapy [4]. However, despite the acknowledged involvement of some MMPs in MS pathogenesis and progression, the evaluation of these enzymes is not routinely recommended for MS diagnosis because their elevation is observed in numerous other diseases as stroke and bacterial and viral infections and even in smokers [5]. Nevertheless, the higher activity of individual MMPs in connection with patients' clinical status can help to predict the risk, diagnosis, or progress of the disease. For example, the MMP-9 serum level does not correlate with the risk of stroke but MMP-9 C(-1562)T polymorphism seems to be significantly associated with risk of stroke in patients with and without type 2 diabetes mellitus [6]. Also remaining MMPs possess the ability to predict the clinical status. The overexpression of MMP-7, MMP-10, and MMP-12 in colon cancer patients' sera correlates with a dismal prognosis [7] and high serum MMP-1 level showed a trend for short overall survival in non-small cell lung cancer patients [8].

The low tissue specificity of isolated MMPs causes that single enzyme may not play a role of a good biomarker. However, some MMPs could be useful constituents of biomarker panels but only in combination with other biochemical parameters. The multiplex panel composed of MMP-7, CA125, CA72-4, and human epididymis protein 4 is suitable for the early detection of ovarian cancer [9]. The simultaneous evaluation of MMP-1, TIMP-1, CD40 ligand, and myeloperoxidase seems to be a novel promising diagnostic panel in timely diagnosis of acute aortic dissection [10]. Also, some products of MMPs catalysis were considered as the potential biomarkers. Citrullinated and MMP-degraded vimentin (VICM) simultaneously and in combination with others markers revealed good potential to differentiate ulcerative colitis form noninflammatory bowel diseases [11].

Finally, last but not least, preanalytical conditions must be taken into account before starting MMPs analysis in body fluids. In fact, if in one hand the release of MMPs during clotting could affect their concentrations [12], on the other hand the use of some calcium-chelating anticoagulants could interfere with MMPs activity [13].

In conclusion, the enzymes from among MMPs evaluated individually cannot be considered as the specific biomarkers of the particular disease or pathological process. However, the sudden change in their body fluid level can act as an alarm siren informing on the upcoming threat which combined with clinical state of the patient may help in the diagnosis, treatment, or prognosis.

*Jacek Kurzepa* *Jacek Kurzepa* *Fatma M. El-Demerdash* *Fatma M. El-Demerdash* *Massimiliano Castellazzi* *Massimiliano Castellazzi*
